# Supplementary material for: Buried treasure in a public repository: Mining mitochondrial genes of 32 annelid species from sequence reads deposited in the Sequence Read Archive (SRA)
Source: PeerJ. 2023 Nov 29;11:e16446. doi: 10.7717/peerj.16446 (PMC10693233; doi:10.7717/peerj.16446)
Supplement: Data S1 [file peerj-11-16446-s001.pdf]

Supplementary data 1. Best-fit substitution model for each gene selected with ModelFinder.

atp6: mtInv+F+R6
atp8: mtZOA+F+R5
cox3: mtZOA+R6
cox2: mtZOA+F+R6
cox1: mtZOA+R5
cytb: mtZOA+R6
nad1: mtART+R6
nad2: mtInv+F+R7
nad3: mtMet+I+G4
nad4l: mtMet+I+G4
nad4: mtInv+F+R7
nad5: mtInv+F+R7
nad6: mtInv+F+I+G4
