# Supplementary material for: Buried treasure in a public repository: Mining mitochondrial genes of 32 annelid species from sequence reads deposited in the Sequence Read Archive (SRA)
Source: PeerJ. 2023 Nov 29;11:e16446. doi: 10.7717/peerj.16446 (PMC10693233; doi:10.7717/peerj.16446)
Supplement: Table S1 — The assembled sequences were deposited as Third Party Data (TPA). [file peerj-11-16446-s002.docx]

Supplementary Table S1. Accession numbers of sequence reads downloaded from NCBI SRA and newly assembled mitochondrial protein-coding gene sequences. The assembled sequences were deposited as Third Party Data (TPA).

| Family | Species | SRR | Data type | atp6 | atp8 | cox1 | cox2 | cox3 | cytb | nad1 | nad2 | nad3 | nad4 | nad4l | nad5 | nad6 |
| --- | --- | --- | --- | --- | --- | --- | --- | --- | --- | --- | --- | --- | --- | --- | --- | --- |
| Opheliidae | *Ophelina acumulata* | SRR10997422 | cDNA | YAAQ01000001 | YAAQ01000002 | YAAQ01000003 | YAAQ01000004 | YAAQ01000005 | YAAQ01000006 | YAAQ01000007 | YAAQ01000008 | YAAQ01000009 | YAAQ01000010 | YAAQ01000011 | YAAQ01000012 | YAAQ01000013 |
|  | *Thoracophelia mucronata* | SRR2017631 | cDNA | - | YAAY01000001 | YAAY01000002 | YAAY01000003 | YAAY01000004 | YAAY01000005 | YAAY01000006 | - | YAAY01000007 | YAAY01000008 | - | YAAY01000009 | - |
| Thalassematidae | *Bonellia viridis* | SRR2017645 | cDNA | YAAG01000001 | YAAG01000002 | YAAG01000003 | YAAG01000004 | YAAG01000005 | YAAG01000006 | YAAG01000007 | YAAG01000008 | YAAG01000009 | YAAG01000010 | YAAG01000011 | YAAG01000012 | YAAG01000013 |
| Trichobranchidae | *Trichobranchus roseus* | SRR11434466 | cDNA | YABA01000001 | YABA01000002 | YABA01000003 | YABA01000004 | YABA01000005 | YABA01000006 | YABA01000007 | YABA01000008 | YABA01000009 | YABA01000010 | - | YABA01000011 | YABA01000012 |
| Arenicolidae | *Arenicola marina* | SRR2005653 | cDNA | YAAE01000001 | YAAE01000002 | YAAE01000003 | YAAE01000004 | YAAE01000005 | YAAE01000006 | YAAE01000007 | YAAE01000008 | YAAE01000009 | YAAE01000010 | YAAE01000011 | YAAE01000012 | YAAE01000013 |
|  | *Abarenicola pacifica* | SRR10997426 | cDNA | YAAB01000001 | YAAB01000002 | YAAB01000003 | YAAB01000004 | YAAB01000005 | YAAB01000006 | YAAB01000007 | YAAB01000008 | YAAB01000009 | YAAB01000010 | YAAB01000011 | YAAB01000012 | YAAB01000013 |
| Scalibregmatidae | *Scalibregma inflatum* | SRR8799334 | cDNA | YAAX01000001 | YAAX01000002 | YAAX01000003 | YAAX01000004 | YAAX01000005 | YAAX01000006 | YAAX01000007 | YAAX01000008 | YAAX01000009 | YAAX01000010 | YAAX01000011 | YAAX01000012 | YAAX01000013 |
| Travisiidae | *Travisia forbesii** | SRR9888046 | cDNA | - | - | YAAZ01000001 | YAAZ01000002 | - | YAAZ01000003 | - | - | - | - | - | YAAZ01000004 |  |
| Aeolosomatidae | *Aeolosoma* sp. | SRR11559519 | cDNA | YAAC01000001 | YAAC01000002 | YAAC01000003 | YAAC01000004 | YAAC01000005 | YAAC01000006 | YAAC01000007 | YAAC01000008 | YAAC01000009 | YAAC01000010 | YAAC01000011 | - | YAAC01000012 |
| Hrabeiellidae | *Hrabeiella periglandulata* | SRR10997424 | cDNA | YAAN01000001 | YAAN01000002 | YAAN01000003 | YAAN01000004 | YAAN01000005 | YAAN01000006 | YAAN01000007 | YAAN01000008 | YAAN01000009 | YAAN01000010 | YAAN01000011 | YAAN01000012 | YAAN01000013 |
| Randiellidae | *Randiella* sp. | SRR10997431 | cDNA | YAAV01000001 | YAAV01000002 | YAAV01000003 | YAAV01000004 | YAAV01000005 | YAAV01000006 | YAAV01000007 | YAAV01000008 | YAAV01000009 | YAAV01000010 | YAAV01000011 | YAAV01000012 | YAAV01000013 |
| Parvidrilidae | *Parvidrilus meyssonnieri* | SRR8799336 | cDNA | - | YAAR01000001 | YAAR01000002 | YAAR01000003 | YAAR01000004 | YAAR01000005 | YAAR01000006 | - | YAAR01000007 | YAAR01000008 | YAAR01000009 | YAAR01000010 | YAAR01000011 |
| Capilloventridae | *Capilloventer australis* | SRR8799324 | cDNA | YAAI01000001 | YAAI01000002 | YAAI01000003 | YAAI01000004 | YAAI01000005 | YAAI01000006 | YAAI01000007 | YAAI01000008 | YAAI01000009 | YAAI01000010 | YAAI01000011 | YAAI01000012 | YAAI01000013 |
| Phreodrilidae | Phreodrilidae sp. A | SRR10997437 | cDNA | YAAS01000001 | YAAS01000002 | YAAS01000003 | YAAS01000004 | YAAS01000005 | YAAS01000006 | YAAS01000007 | YAAS01000008 | YAAS01000009 | YAAS01000010 | YAAS01000011 | YAAS01000012 | YAAS01000013 |
| Naididae | *Albanidrilus* sp | SRR10997452 | cDNA | YAAD01000001 | YAAD01000002 | YAAD01000003 | YAAD01000004 | YAAD01000005 | YAAD01000006 | YAAD01000007 | YAAD01000008 | YAAD01000009 | YAAD01000010 | YAAD01000011 | YAAD01000012 | YAAD01000013 |
|  | *Chaetogaster diaphanus* | SRR10997419 | cDNA | YAAJ01000001 | YAAJ01000002 | YAAJ01000003 | YAAJ01000004 | YAAJ01000005 | YAAJ01000006 | YAAJ01000007 | YAAJ01000008 | YAAJ01000009 | YAAJ01000010 | YAAJ01000011 | YAAJ01000012 | YAAJ01000013 |
|  | *Bathydrilus rohdei* | SRR8799332 | cDNA | YAAF01000001 | - | YAAF01000002 | YAAF01000003 | YAAF01000004 | YAAF01000005 | YAAF01000006 | YAAF01000007 | YAAF01000008 | YAAF01000009 | YAAF01000010 | YAAF01000011 | YAAF01000012 |
|  | *Olavius* sp. | SRR8799329 | cDNA | YAAP01000001 | YAAP01000002 | YAAP01000003 | YAAP01000004 | YAAP01000005 | YAAP01000006 | YAAP01000007 | YAAP01000008 | YAAP01000009 | YAAP01000010 | YAAP01000011 | YAAP01000012 | YAAP01000013 |
|  | *Potamothrix* nr *heuscheri* | SRR10997432 | cDNA | YAAT01000001 | YAAT01000002 | YAAT01000003 | YAAT01000004 | YAAT01000005 | YAAT01000006 | YAAT01000007 | YAAT01000008 | YAAT01000009 | YAAT01000010 | YAAT01000011 | YAAT01000012 | YAAT01000013 |
|  | *Rhyacodrilus pigueti* | SRR8799325 | cDNA | YAAW01000001 | YAAW01000002 | YAAW01000003 | YAAW01000004 | YAAW01000005 | YAAW01000006 | YAAW01000007 | YAAW01000008 | YAAW01000009 | YAAW01000010 | YAAW01000011 | YAAW01000012 | YAAW01000013 |
| Propappidae | *Propappus volki* | SRR5353250 | cDNA | YAAU01000001 | - | YAAU01000002 | YAAU01000003 | YAAU01000004 | YAAU01000005 | YAAU01000006 | YAAU01000007 | YAAU01000008 | YAAU01000009 | - | YAAU01000010 | YAAU01000011 |
| Enchytraeidae | *Grania simonae* | SRR10997449 | cDNA | YAAL01000001 | YAAL01000002 | YAAL01000003 | YAAL01000004 | YAAL01000005 | YAAL01000006 | YAAL01000007 | YAAL01000008 | YAAL01000009 | YAAL01000010 | YAAL01000011 | YAAL01000012 | YAAL01000013 |
|  | *Enchytraeus crypticus* | SRR10997417 | cDNA | YAAK01000001 | YAAK01000002 | YAAK01000003 | YAAK01000004 | YAAK01000005 | YAAK01000006 | YAAK01000007 | YAAK01000008 | YAAK01000009 | YAAK01000010 | - | YAAK01000011 | YAAK01000012 |
| Family | Species | SRR | Data type | atp6 | atp8 | cox1 | cox2 | cox3 | cytb | nad1 | nad2 | nad3 | nad4 | nad4l | nad5 | nad6 |
| Lumbriculidae | *Lumbriculus variegatus* | SRR8842488 | DNA | BR001868 | - | BR001869 | BR001870 | BR001871 | BR001872 | BR001873 | BR001874 | BR001875 | BR001876 | BR001877 | BR001878 | BR001879 |
|  | *Kincaidiana* sp. | SRR10997445 | cDNA | YAAO01000001 | YAAO01000002 | YAAO01000003 | YAAO01000004 | YAAO01000005 | YAAO01000006 | YAAO01000007 | YAAO01000008 | YAAO01000009 | YAAO01000010 | - | YAAO01000011 | YAAO01000012 |
| Branchiobdellidae | *Cirrodrilus suzukii* | SRR8842480 | DNA | BR001834 | BR001835 | BR001836 | BR001837 | BR001838 | BR001839 | BR001840 | BR001841 | BR001842 | BR001843 | - | BR001844 | BR001845 |
|  | *Branchiobdella kobayashii* | SRR8799326 | cDNA | YAAH01000001 | YAAH01000002 | YAAH01000003 | YAAH01000004 | YAAH01000005 | YAAH01000006 | YAAH01000007 | YAAH01000008 | YAAH01000009 | YAAH01000010 | YAAH01000011 | YAAH01000012 | YAAH01000013 |
|  | *Holtodrilus truncatus* | SRR8842481 | DNA | BR001856 | BR001857 | BR001858 | BR001859 | BR001860 | BR001861 | BR001862 | BR001863 | BR001864 | BR001865 | - | BR001866 | BR001867 |
|  | *Bdellodrilus illuminatus* | SRR8842477 | DNA | BR001822 | - | BR001823 | BR001824 | BR001825 | BR001826 | BR001827 | BR001828 | BR001829 | BR001830 | BR001831 | BR001832 | BR001833 |
|  | *Triannulata magna* | SRR8842482 | DNA | BR001880 | - | BR001881 | BR001882 | BR001883 | BR001884 | BR001885 | BR001886 | BR001887 | BR001888 | BR001889 | BR001890 | BR001891 |
| Cylicobdellidae | Cylicobdellidae sp. | SRR8842484 | DNA | BR001846 | - | BR001847 | BR001848 | BR001849 | BR001850 | BR001851 | - | BR001852 | BR001853 | - | BR001854 | BR001855 |
| Haemopidae | *Haemopis sanguisuga* | SRR10997447 | cDNA | YAAM01000001 | - | YAAM01000002 | YAAM01000003 | YAAM01000004 | YAAM01000005 | YAAM01000006 | YAAM01000007 | YAAM01000008 | YAAM01000009 | - | YAAM01000010 | YAAM01000011 |

*Gene sequences were not used for phylogenetic analysis.
