# Supplementary material for: Buried treasure in a public repository: Mining mitochondrial genes of 32 annelid species from sequence reads deposited in the Sequence Read Archive (SRA)
Source: PeerJ. 2023 Nov 29;11:e16446. doi: 10.7717/peerj.16446 (PMC10693233; doi:10.7717/peerj.16446)
Supplement: Table S2 [file peerj-11-16446-s003.docx]

Supplementary Table 2. The length of genes that were partly (incompletely) assembled.

| Species | Length* | Locus | % assembled** |
| --- | --- | --- | --- |
| *Arenicola marina* | <1..>1544 | cox1 | 99 |
| *Arenicola marina* | <1..1125 | cytb | 99 |
| *Bathydrilus rohdei* | <1..1488 | cox1 | 97 |
| *Bathydrilus rohdei* | 1..>777 | cox3 | 99 |
| *Bathydrilus rohdei* | 1..>453 | nad6 | 97 |
| *Bonellia viridis* | 1..>159 | atp8 | 99 |
| *Bonellia viridis* | 1..>1529 | cox1 | 99 |
| *Bonellia viridis* | <1..1113 | cytb | 99 |
| *Bonellia viridis* | 1..>852 | nad2 | 86 |
| *Bonellia viridis* | 1..>1692 | nad5 | 99 |
| *Chaetogaster diaphanus* | <1..699 | nad2 | 71 |
| *Chaetogaster diaphanus* | <1..1485 | nad5 | 90 |
| *Cirrodrilus suzukii* | <1..>1419 | nad5 | 86 |
| Cylicobdellidae sp. | 1..>1138 | nad5 | 69 |
| Cylicobdellidae sp. | 1..>279 | nad6 | 60 |
| *Enchytraeus crypticus* | <1..795 | nad4 | 59 |
| *Enchytraeus crypticus* | 1..>1607 | nad5 | 97 |
| *Enchytraeus crypticus* | 1..>465 | nad6 | 99 |
| *Haemopis sanguisuga* | 1..>987 | nad2 | 99 |
| *Haemopis sanguisuga* | <1..345 | nad3 | 99 |
| *Haemopis sanguisuga* | <1..1104 | nad4 | 67 |
| *Haemopis sanguisuga* | <1..1605 | nad5 | 97 |
| *Holtodrilus truncatus* | <1..468 | atp6 | 68 |
| *Holtodrilus truncatus* | 1..>615 | nad2 | 62 |
| *Hrabeiella periglandulata* | <1..294 | nad3 | 84 |
| *Hrabeiella periglandulata* | <1..1563 | nad5 | 95 |
| *Kincaidiana* sp. | <1..792 | nad1 | 86 |
| *Lumbriculus variegatus* | <1..198 | nad4l | 68 |
| *Lumbriculus variegatus* | <1..>1500 | nad5 | 91 |
| *Olavius* sp. | 1..>1530 | cox1 | 99 |
| *Ophelina acuminata* | 1..>984 | nad2 | 99 |
| *Ophelina acuminata* | 1..>294 | nad4l | 99 |
| *Ophelina acuminata* | <1..1695 | nad5 | 99 |
| Phreodrilidae sp. | 1..>1719 | nad5 | 99 |
| *Propappus volki* | 1..>933 | nad1 | 99 |
| *Randiella* sp. | 1..>777 | cox3 | 99 |
| Species | Length* | Locus | % assembled** |
| *Scalibregma inflatum* | 1..>159 | atp8 | 99 |
| *Scalibregma inflatum* | 1..>1542 | cox1 | 99 |
| *Thoracophelia mucronata* | 1..>777 | cox3 | 99 |
| *Thoracophelia mucronata* | <1..1143 | cytb | 99 |
| *Thoracophelia mucronata* | 1..>351 | nad3 | 99 |
| *Travisia forbesii* | 1..>1110 | cytb | 98 |
| *Travisia forbesii* | <1..>481 | nad5 | 29 |
| *Triannulata magna* | 1..>459 | nad6 | 98 |
| *Trichobranchus roseus* | 1..>1008 | nad2 | 99 |
| *Trichobranchus roseus* | 1..>1362 | nad4 | 83 |

*<: anteriorly incomplete, >: posteriorly incomplete.

**The ratio of assembled length to the average length of the dataset for phylogenetic analysis. When assembled length was longer than the average length (≥100%), it was treated as 99%.
